# Draft Genome Sequence of Carbaryl-Degrading Soil Isolate *Pseudomonas* sp. Strain C5pp

**DOI:** 10.1128/genomeA.00526-16

**Published:** 2016-06-09

**Authors:** Vikas D. Trivedi, Pramod Kumar Jangir, Rakesh Sharma, Prashant S. Phale

**Affiliations:** a Department of Biosciences and Bioengineering, Indian Institute of Technology Bombay, Powai, Mumbai, India; b CSIR-Institute of Genomics and Integrative Biology, New Delhi, India

## Abstract

We report the draft genome sequence of carbaryl-degrading *Pseudomonas* sp. strain C5pp. Genes encoding salicylate and gentisate metabolism, large amounts of oxygenase, nitrogen metabolism, and heavy metal tolerance were identified. The sequence will provide further insight into the biochemical and evolutionary aspects of carbaryl degradation.

## GENOME ANNOUNCEMENT

Carbaryl, 1-naphthyl *N*-methylcarbamate, is used as a broad-spectrum insecticide. It is highly toxic for aquatic invertebrates, amphibians, bees, and earthworms (1, 2). Soil isolate *Pseudomonas* sp. strain C5pp metabolizes carbaryl to the intermediate tricarboxylic acid (TCA) cycle (3–5). To date, there is no genome sequence data available from any of the carbaryl-degrading organisms.

Soil isolate *Pseudomonas* sp. strain C5pp (referred to as strain C5pp herein, earlier referred to as strain C5) was grown on carbaryl as described in reference 3. The genomic DNA was isolated using an Ultra-Clean Microbial DNA isolation kit (MoBio, USA) and sequenced using the whole-genome shotgun 454, GS FLX+ strategy, which generated 249,174 raw reads which were assembled using Newbler (version 2.9) into 97 large contigs (≥500 bp) and 34 smaller contigs (<500 bp). The draft genome of strain C5pp is estimated to be 6.15 Mb in size (26× coverage) with 62.65% G+C content. The overall contiguity of the assembly is good, with an *N*_50_ of 147.9 Kbp, and the longest assembled contig is 345.7 Kbp in length. Genome annotation was performed with the NCBI-Prokaryotic Genome Annotation Pipeline (PGAP) and Rapid Annotation using Subsystems Technology (RAST) server (6). The strain C5pp genome contains 5,506 protein coding sequences; rRNA genes: 5S (2 copies), 16S (1 copy), and 23S (1 copy); and 68 tRNA genes.

The draft genome revealed the presence of 49 oxygenase genes involved in aromatic as well as various cellular metabolic processes. Genes involved in salicylate to gentisate (“middle”) and gentisate to TCA cycle (“lower” segment) of the carbaryl pathway were identified. Additionally, oxygenase genes involved in benzoate, protocatechuate, catechol, phenylacetate, and *p*-hydroxyphenylacetate metabolism were identified. Strain C5pp shows good growth on benzoate, *p*-hydroxybenzoate, salicylate, gentisate, protocatechuate (3, 7), and phenylacetate as carbon sources (0.1%), indicating that oxygenases are functional in strain C5pp. The draft genome of strain C5pp also showed the presence of 50 genes associated with nitrogen metabolism. This includes the genes for cyanate hydrolysis, nitrosative stress, nitrate and nitrite ammonification, and ammonia assimilation. A few of these genes may have a role in channeling the nitrogen from the carbaryl molecule into the carbon-nitrogen cycle. Putative genes involved in heavy metal tolerance (copper, zinc, cobalt, and cadmium) were identified.

The property for aromatic compound degradation is acquired by horizontal gene transfer (HGT) events involving genomic islands (GIs), transposases, integrons, etc. (8). Thirty-six GIs were predicted in the draft genome of strain C5pp by SIGI-HMM using IslandViewer29. Overall, 43 mobile genetic elements (transposases and integrases) were identified.

The genome analysis indicates the ability of *Pseudomonas* sp. strain C5pp to degrade pools of aromatic compounds and heavy metal tolerance, thus allowing the strain to be used as a potential candidate for bioremediation of contaminated environment. Further studies will be directed toward the functional analysis of the carbaryl metabolic pathway genes as well as the probable HGT events involved in acquiring the carbaryl degradation trait.

### Nucleotide sequence accession number.

This draft genome sequence has been deposited at NCBI with the GenBank accession number JWLN00000000.
